# QSAR Modeling of Acute Aquatic Toxicity of Ethoxylated Alcohol Mixtures, and Estimation of Solubility Cut-Offs

**DOI:** 10.1021/acs.chemrestox.5c00561

**Published:** 2026-08-28

**Authors:** David W. Roberts, M. Teresa García, Isabel Ribosa

**Affiliations:** † School of Pharmacy and Biomolecular Sciences, 4589 Liverpool John Moores University, Liverpool L3 3AF, United Kingdom; ‡ Department of Surfactants and Nanobiotechnology, Institute for Advanced Chemistry of Catalonia (IQAC−CSIC), 08034 Barcelona, Spain

## Abstract

Commercial nonionic surfactants are multicomponent mixtures, typically derived from ethoxylation of a primary alcohol that may be a mixture of homologues and isomers with a range of degrees of ethoxylation. *Daphnia magna* and *Vibrio fischeri* toxicity data have been obtained for several nonionic surfactants, covering a wide range of average degrees of ethoxylation and parent alcohol structure, including both narrow-range and broad-range ethoxamer distributions. These data are analyzed by means of a general narcosis quantitative structure–activity relationship (QSAR) based on calculated log *P* values together with mixture toxicity equations. In some cases not all components are sufficiently soluble to make their full toxic contributions. An approach for identifying such components and for taking their reduced toxic contributions into account is derived from previously published structure-solubility relationships and validated against the experimental toxicity data.

## Graphical abstract

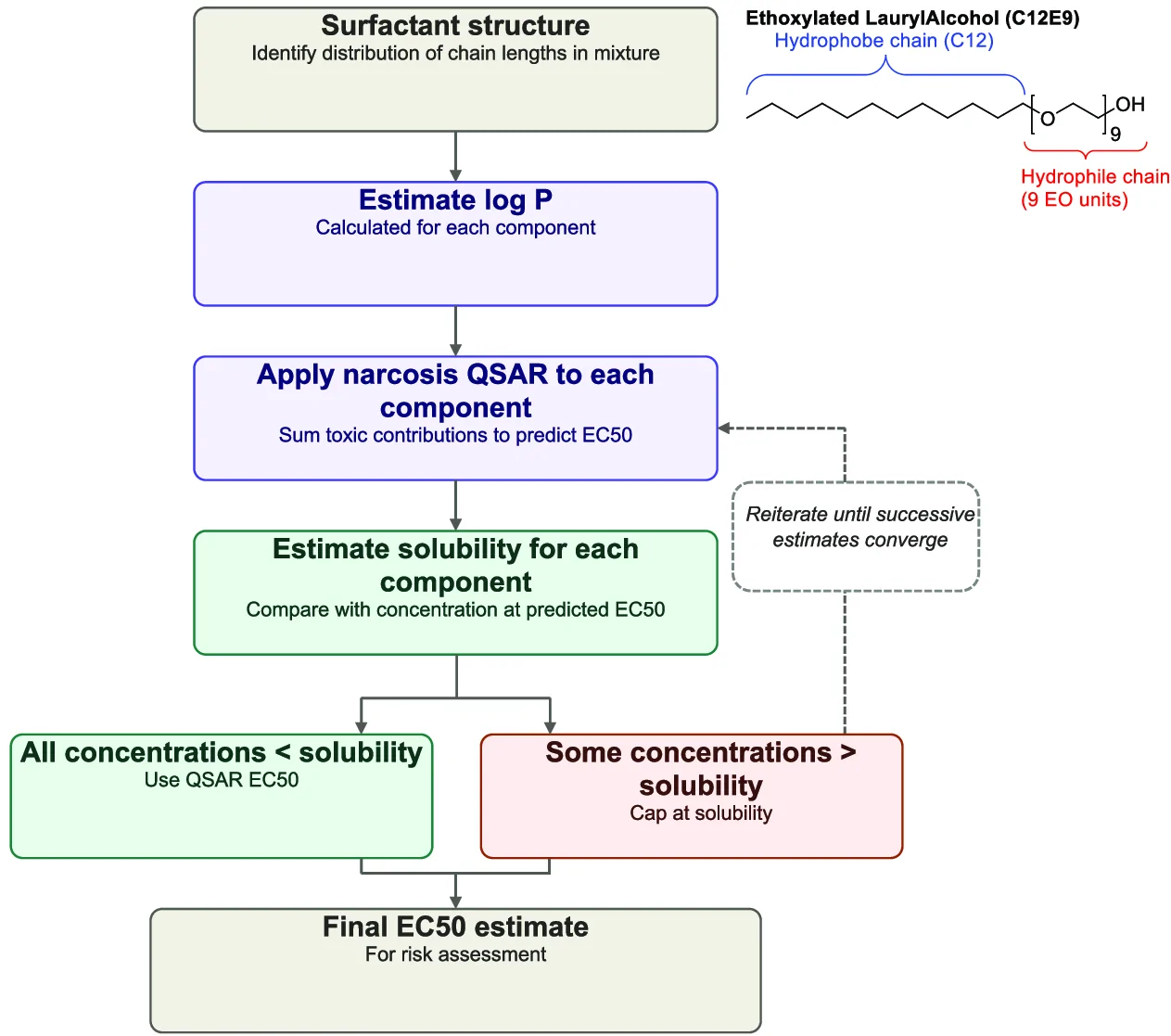

## Introduction

The application of QSAR to the aquatic toxicity of nonionic surfactants, of the types widely used in large tonnage consumer products such as washing powders, fabric washing liquids and dish washing liquids, is the topic of this paper. These surfactants are manufactured by reaction of primary alcohols with ethylene oxide (EO)

$$\mathrm{ROH}+nEO\to R{\left({\mathrm{OCH}}_{2}{\mathrm{CH}}_{2}\right)}_{n}\mathrm{OH}$$

The commercial ethoxylated alcohols are multicomponent mixtures, with a range of degrees of ethoxylation *n* and in some cases a range of homologues and isomers for the parent alcohol ROH. The manufacturing routes for these surfactants and the structures of the ones considered here are shown in Scheme 1.

**Scheme 1:**
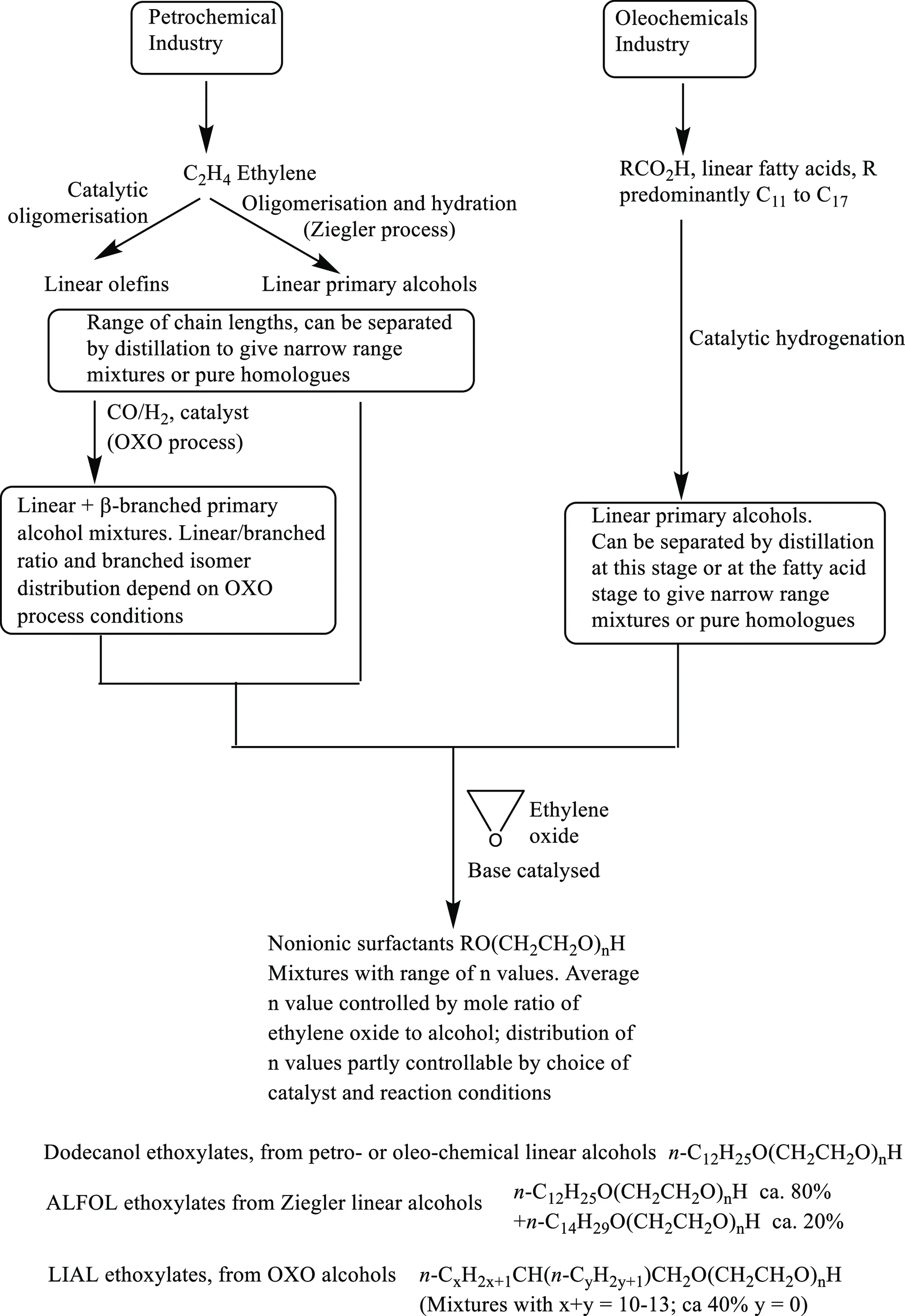
Manufacturing Routes and Structures for Ethoxylated Alcohol Surfactants

Single component ethoxylated alcohols can be synthesized on laboratory scale but are not used in commercial detergent products.

The aquatic toxicity of many surfactants can be predicted from QSAR equations using hydrophobicity parameters in the same way as for chemically unreactive nonsurfactant organic chemicals.^1–3^

The octanol/water partition coefficient, sometimes represented as *P* (for Partition) and sometimes as *K*_o/w_ (reflecting that partitioning is an equilibrium process), normally used as its logarithm, has proved to be a very useful hydrophobicity parameter in quantitative structure–activity relationships (QSAR). In ecotoxicology QSAR, particularly when experimentally determined partition coefficients are used, log *K*_o/w_ seems to be preferred to log *P* by many authors. Because the partition coefficients used in this paper are all calculated, by a method referred to by its authors^4^ as applying for log *P*, we use the log *P* terminology here.

Log *P* can be difficult to measure for surfactants, and indeed it has been argued^5^ that measured interfacial properties are more appropriate for use in surfactant toxicology QSARs. However, it has been shown that calculated log *P* values for surfactants can be applied successfully not only for surfactant toxicity QSARs^6^ but also for modeling surfactant properties such as critical micelle concentration.^7^ Surfactant toxicity QSARs based on log *P* can be compared directly with QSARs for nonsurfactants with known mechanisms of action, and in this way, together with mixture toxicity studies, it has been shown that, in their acute aquatic toxicity, anionic and nonionic surfactants act by polar and general narcosis, respectively.^5–9^

In a 1998 paper by Vaes et al.^10^ it was argued that the difference between polar and general narcosis is an artifact that disappears when log *P* is replaced by a liposome-water partition coefficient and this claim can still be found in the more recent literature, e.g., ref 11. However, the mechanistic difference between polar and general narcosis has been discussed in detail and, based on the evidence of mixture toxicity studies and fish acute toxicity syndrome findings, in addition to regression analysis, shown to be real.^12^ This is confirmed in a more recent experimental study by Droge et al.^13^

Commercial nonionic surfactants derived from ethoxylation of primary alcohols can contain particularly large numbers of components, since the parent alcohol may consist of several isomers and homologues (as few as 2, for example in the case of a coconut or Ziegler alcohol or as many as 26, for example in the case of a C12–15 OXO-alcohol) and corresponding to each of these alcohol components there is a range of components ethoxylated to different degrees. For a given average degree of ethoxylation, by selection of the reaction conditions it is possible to produce a relatively broad range or a relatively narrow range ethoxamer distribution.^14^

For relatively low average degrees of ethoxylation (≤ca. 7), it has been shown^2^ that the mixture toxicity calculations can be simplified by treating the mixture as though each of the parent alcohols ROH had been converted to a hypothetical single derivative having the same number (which may be a noninteger) of EO units as the overall average. However, with higher average degrees of ethoxylation this simplifying approximation becomes less reliable, tending to underestimate the extent to which the overall toxicity will be dominated by the contributions of the small amounts of lower ethoxamers present.

In this paper we show how the mixture toxicity approach can be extended so as to be applicable to higher degrees of ethoxylation, using a previously published toxicity data set^15,16^ for three different alcohols, each ethoxylated to various degrees ranging from 0.4 EO (i.e., an average of 0.4 EO units) to 19.8 EO and including both broad range distributions (BRD) and narrow range distributions (NRD) of ethoxamers. Reference 15 is in a conference proceedings book and may not be easily accessible to some readers. Reference 16 presents a peer-reviewed account of the same toxicity data, reporting the influence of narrow- versus broad-range ethoxamer distributions on the acute aquatic toxicity of these ethoxylated alcohols.

## Materials and Methods

### Ethoxylated Alcohol Compositions

The ethoxylated alcohols were synthesized on the laboratory scale from three parent alcohols:

*n*-Dodecanol, of 98% purity.

ALFOL 12–14, a commercial Ziegler alcohol consisting of *n*-dodecanol (78.5%) and *n*-tetradecanol (20.7%).

LIAL 125, a commercial C12–15 OXO primary alcohol consisting of ca. 40% linear and 60% 2-branched isomers. The general structure is R**R**CHCH_2_OH where R is alkyl and **R** is alkyl or hydrogen. The isomer and homologue distribution, based on chromatographic analysis of a different sample of LIAL 125 at Unilever Research, is shown in Table 1.

**Table 1:** LIAL 125 Isomer and Homologue Distribution^a^

C–No	Br	%	C–No	Br	%
12	0	10.5	14	1	4.4
12	1	3.5	14	2	2.5
12	2	1.8	14	3	2.8
12	3	1.9	14	4	3.4
12	4	1.5	14	5	3.7
12	5	1.6	14	6	1.8
13	0	11.2	15	0	5.8
13	1	5.0	15	1	2.3
13	2	2.8	15	2	1.3
13	3	3.3	15	3	1.6
13	4	3.8	15	4	2.1
13	5	4.3	15	5	2.4
14	0	12.2	15	6	2.2

a Br = length of shorter branch R or **R** in R**R**CHCH_2_OH.

The ethoxylation of these alcohols to produce both broad range distribution (BRD) and narrow range distribution (NRD) ethoxylates, has been described previously.^14^ The ethoxamer distributions were determined chromatographically (using gas chromatography, GC, for lower EO materials and liquid chromatography, LC, for higher EO materials) as described by Voelkel et al.^14^ Some of these distributions have been shown previously.^15,16^

Full details of the ethoxamer distributions and toxicity data (from ref 15) are provided in the Supporting Information document SI1.

### Calculation of Toxicity Values

The method used is an adaptation of the previously described method.^2^ In brief, log *P* is calculated as follows:

By the Leo and Hansch method^4^

$$\log P\;\mathrm{for}\;n‐C_{12}H_{25}\mathrm{OH is}\;5.19$$

$$\log P\;\mathrm{for}\;n‐C_{12}H_{25}{\mathrm{OCH}}_{2}{\mathrm{CH}}_{2}\mathrm{OH is}\;5.23$$

From these two reference compounds, log *P* values are derived for other homologues, isomers and ethoxamers:

For each additional EO unit, 0.10 is subtracted. Note that, as discussed previously^1^ the first EO unit added to an alcohol increases the log *P* value slightly, whereas the log *P* increments for further EO units are negative.

For each increment in alkyl group carbon number, 0.54 is added.

For branching in the alkyl group, if *B* is the carbon number of the shorter of the R and **R** groups in R**R**CHCH_2_OH, 1.44 log­(1 + *B*) is subtracted.

This manual method differs slightly from the present computerized *C* log *P* method although both derive from the same origin (*vide infra*).

From the log *P* value, EC_50_ is calculated for each component using the Könemann general narcosis equation^17^

1 $$\log\left(1/{\mathrm{EC}}_{50}\right)\left(\mathrm{mole}/l\right)=0.87\log P+1.13$$

Although originally developed for fish toxicity, the Könemann equation has been found to be applicable with little error to many other aquatic organisms, including *Daphnia magna*.^1^

For each component in the mixture, the weight fraction is divided by the EC_50_, in mg/L units, to give the toxic contribution. The sum of the toxic contributions gives the reciprocal of the EC_50_ of the mixture.

These calculations are conveniently carried out by means of a spreadsheet, and for illustration the spreadsheet used for *n*-dodecanol-E1.1 (NRD) is shown in Table 2.

**Table 2:** Spreadsheet for Fish Toxicity Calculation of Dodecanol-E1.1 NRD^a^ ^,^ ^b^

EC_50_ is in units of mg/L						
	A	B	C	D	E	F
1						
2	**E No.**	**%**	**M**	**log *P***	**EC_50_**	**Tox. cont.**
3	0	37.7	186	5.19	0.42	0.90
4	1	22.5	230	5.23	0.48	0.47
5	2	19.2	274	5.13	0.70	0.27
6	3	12.3	318	5.03	0.99	0.12
7	4	5.7	362	4.93	1.38	0.04
8	5	1.9	406	4.83	1.89	0.01
9	6	0.4	450	4.73	2.56	0.00
10	7	0.1	494	4.63	3.43	0.00
11	8	1.0	538	4.53	4.57	0.00
12	**Overall EC_50_**					0.55

a Formulas: Column C: row 3 entered manually; C4 = C3 + 44, etc. Column D: rows 3 and 4 entered manually; D5 = D4–0.10, etc. Column E: E3 = 10∧(3+ log­(C3)-0.87 × D3–1.13), etc. Column F (rows 3–11): F3 = B3/(100 × E3), etc. F12: = 1/SUM­(F3···F11)

b Legend: Column A lists the ethoxamers present in the mixture, and column B lists their percentages in the mixture. These data are entered manually. Column C lists the molecular weights; these are calculated in the spreadsheet, starting from the molecular weight of the parent alcohol (186) by addition of 44 for each EO unit. Column D lists the log *P* values; those for the parent alcohol and the E1 ethoxamer are entered manually and those for the E > 1 ethoxamers are calculated in the spreadsheet by subtraction of 0.10 for each additional EO unit. Column E contains the formula for calculation of EC_50_ for each ethoxamer, by application of the Könemann equation; the figures displayed are the results of these calculations. Column F (rows 3–11) contains the formula for calculation of toxic contribution for each ethoxamer; the weight fraction (figure from column B, divided by 100) is divided by the EC_50_ (result of the calculation in column E). Finally, cell F 12 contains the formula for summing the toxic contributions and taking the reciprocal of the sum; the result which is displayed is the EC_50_ of the mixture.

For the ALFOL ethoxylates, there are two parent alcohols to consider. However, it is assumed that the linear C14 alcohol and its ethoxyates do not contribute significantly to the toxicity because the parent alcohol and its lower ethoxamers, which are intrinsically the most toxic components, are insufficiently soluble. Therefore, the ALFOL derivatives are treated as though they were n-dodecanol ethoxylates, except that the weight fraction of each ethoxamer is multiplied by 0.785, this being the fraction of C12 homologue in the parent alcohol.

For the LIAL ethoxylates, the calculation is conveniently done using two spreadsheets. In the first, using the known homologue/isomer distribution in the parent alcohol, the EC_50_ values are calculated for the parent alcohol mixture, for a hypothetical mixture of E1 ethoxamers and for hypothetical E2, E3··· up to E20 single ethoxamer mixtures. Ethoxamers above E20 are neglected, on the assumption that they will not contribute significantly to the overall toxicity. It is assumed that linear C14 and C15 alcohols and ethoxamers do not contribute significantly to the toxicity (see comments above for ALFOL ethoxylates). In a second spreadsheet, the ethoxamer distributions and the EC_50_ values for the hypothetical single ethoxamer mixtures are used to calculate the EC_50_ value for each ethoxamer mixture.

An implicit assumption in the LIAL and ALFOL calculations is that identical ethoxamer distributions arise from each component in the parent alcohol.

## Results and Discussion

### Toxicity to *Daphnia*

Table 3 compares the EC_50_ values calculated using eq 1, the Könemann equation for fish, against the observed EC_50_ values in *Daphnia* tests, for all of the ethoxylated alcohols. It can be seen that the toxicity is quite well predicted by the calculated values in spite of all the simplifications previously made. Thus, the assumptions made can be considered substantially correct. The major discrepancy is in the case of dodecanol-E16.7 (NRD), whose observed EC_50_ is about 5 times the calculated value. The case of dodecanol-E11.5 (NRD) can be considered a minor discrepancy, although not sufficiently anomalous to justify treating it as an outlier. We will return to these discrepancies in the section “Estimation and Application of Solubility Cut-Offs”. Figure 1 shows a plot of log EC_50_ (observed) vs log EC_50_ (calculated). Regression analysis for the whole data set except dodecanol-E16.7 (NRD) gives the QSAR equation

2 $$\log{\mathrm{EC}}_{50}\left(\mathrm{Daphnia},\mathrm{obs}\right)=0.88\left(\pm 0.04\right)\log{\mathrm{EC}}_{50}\left(\mathrm{Fish},\mathrm{calc}\right)+0.20\left(\pm 0.03\right)n=35,,R^{2}=0.928,\;R^{2}\left(\mathrm{adj}\right)=0.926,\;s=0.13,\;F=428.1$$

**Table 3:** *Daphnia* Toxicity

Surfactant	EC_50_ obs	EC_50_ calc	Surfactant	EC_50_ obs	EC_50_ calc
Dodecanol BRD			ALFOL BRD		
1.1	1.1	0.56	2.4	1.4	1.03
3.3	1.9	1.02	4.3	2.0	1.69
5.4	2.8	2.66	9.7	4.2	5.24
6.6	3.2	3.32	13.2	14	9.56
8.5	6.9	5.80	15.3	15	14.56
11.2	18	11.05	ALFOL NRD		
14.8	41	27.67	2.5	1.5	1.08
Dodecanol NRD			4.2	1.8	1.74
1.1	1.0	0.55	10.8	4.5	5.10
3.4	1.6	1.10	14.7	20	20.90
5.4	3.4	1.94	16.5	38	55.49
6.4	3.8	1.84			
7.9	8.9	3.77			
11.5	29	8.25			
16.7	240	43.04			

Surfactant	EC_50_ obs	EC_50_ calc	Surfactant	EC_50_ obs	EC_50_ calc
LIAL 125 BRD			LIAL 125 NRD		
0.4	0.8	0.43	0.4	0.9	0.51
3.1	1.3	0.94	2.7	1.1	0.78
4.8	2.4	1.27	4	2.2	0.99
5.1	2.4	1.36	6.2	2.4	1.77
13.6	5.9	4.11	13.4	5.9	3.95
18.7	8.0	9.20	19.8	13	7.11

**Figure 1:**
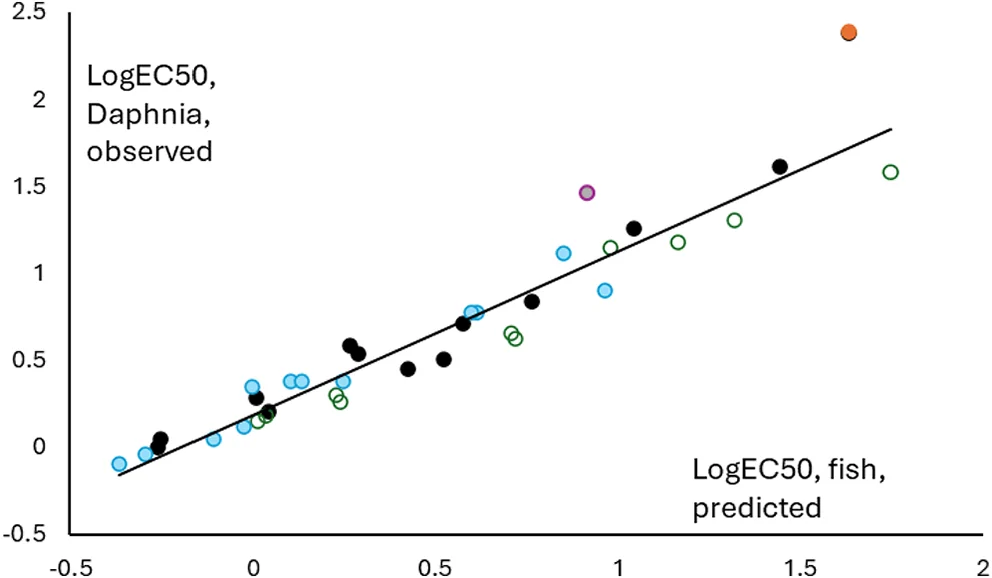
QSAR plots for *Daphnia* (*outlier excluded). Legend: black—ethoxylated Dodecanol; open—ethoxylated ALFOL; blue—ethoxylated LIAL 125; red—outlier (Dodecanol E16.7 NRD), not included in regression; gray—marginal outlier (Dodecanol E11.5 NRD), included in regression.

This regression equation is, within the error limits, identical to that previously reported^2^ for a different set of commercial ethoxylated alcohols, all of which had average degrees of ethoxylation below 7

3 $$\log{\mathrm{EC}}_{50}\left(\mathrm{Daphnia},\mathrm{obs}\right)=0.91\left(\pm 0.10\right)\log{\mathrm{EC}}_{50}\left(\mathrm{Fish},\mathrm{calc}\right)-0.00\left(\pm 0.07\right)$$

This further supports the applicability of the Könemann general narcosis equation to *Daphnia* toxicity of nonionic surfactants (Figure 1).

Some differences do, however, become apparent when we carry out separate regression for each of the three parent alcohols:

Ethoxylated Dodecanol (excluding Dodecanol-E16.7(NRD))

4 $$\log{\mathrm{EC}}_{50}\left(\mathrm{Daphnia},\mathrm{obs}\right)=0.98\left(\pm 0.09\right)\log{\mathrm{EC}}_{50}\left(\mathrm{Fish},\mathrm{calc}\right)+0.18\left(\pm 0.06\right)n=13,,R^{2}=0.922,\;R^{2}\left(\mathrm{adj}\right)=0.915,\;s=0.15,\;F=129.5$$

Ethoxylated ALFOL 12-14

5 $$\log{\mathrm{EC}}_{50}\left(\mathrm{Daphnia},\mathrm{obs}\right)=0.88\left(\pm 0.05\right)\log{\mathrm{EC}}_{50}\left(\mathrm{Fish},\mathrm{calc}\right)+0.10\left(\pm 0.05\right)n=10,,R^{2}=0.971,R^{2}\left(\mathrm{adj}\right)=0.963,s=0.09,F=304.2$$

Ethoxylated LIAL 125

6 $$\log{\mathrm{EC}}_{50}\left(\mathrm{Dapnia},\mathrm{obs}\right)=0.87\left(\pm 0.07\right)\log{\mathrm{EC}}_{50}\left(\mathrm{Fish},\mathrm{calc}\right)+0.22\left(\pm 0.03\right)n=12,,R^{2}=0.948,R^{2}\left(\mathrm{adj}\right)=0.943,s=0.09,F=181.3$$

These differences may be the result of our treating the >C12 linear components of the ALFOL and LIAL ethoxylates as making no contribution to the toxicity, based on the simplifying assumption of insolubility. To the extent that these neglected components do contribute to the toxicity, the calculated fish EC_50_s would overestimate the Daphnia EC_50_s, leading to lower values for the log EC_50_(fish, calc) coefficient in the regression equations.

### Toxicity to *Vibrio fischeri*

Table 4 shows the *V. fischeri* toxicity data (IC_50_). The IC_50_ values are substantially different from the *Daphnia* EC_50_ values shown in Table 3, and hence it is clear that *Daphnia* EC_50_ values, calculated or observed, cannot directly predict *V. fischeri* IC_50_ values. However, as shown in Figure 2, log IC_50_ values are linearly correlated with the calculated log EC_50_ values based on the Könemann equation.

**Figure 2:**
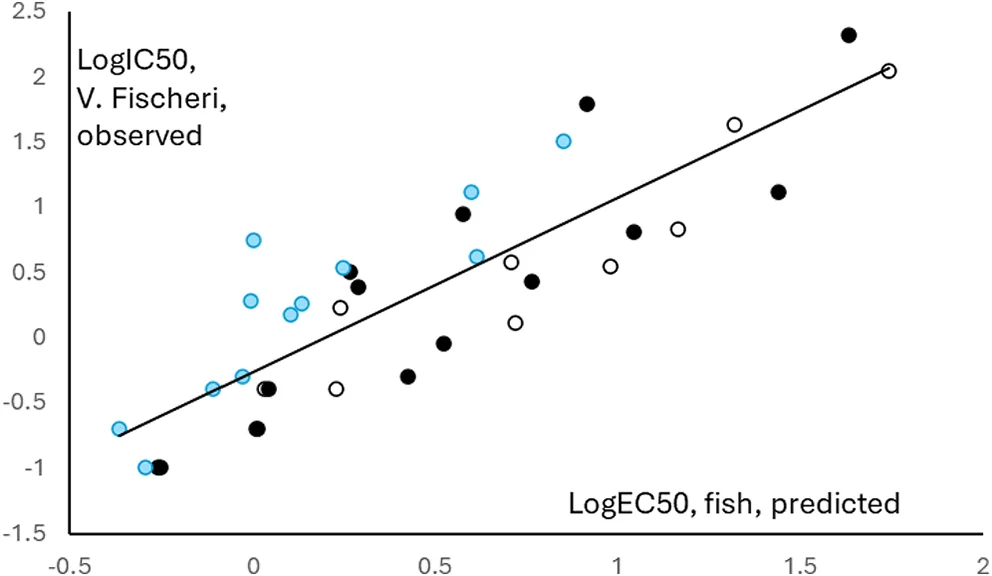
QSAR plots for *Photobacterium phosphoreum*. Black circles, ethoxylated Dodecanol; open circles, ethoxylated ALFOL 12–14; blue circles, ethoxylated LIAL125. Regression line shown is for the full set of data.

**Table 4:** *V. fischeri* Toxicity

Surfactant	IC_50_ obs	EC_50_ calc	Surfactant	IC_50_ obs	EC_50_ calc
Dodecanol BRD			ALFOL BRD		
1.1	0.1	0.56	2.4	0.2	1.03
3.3	0.2	1.02	4.3	0.4	1.69
5.4	0.5	2.66	9.7	1.3	5.24
6.6	0.9	3.32	13.2	3.5	9.56
8.5	2.7	5.80	15.3	6.8	14.56
11.2	6.4	11.05	ALFOL NRD		
14.8	13	27.67	2.5	0.4	1.08
Dodecanol NRD			4.2	1.7	1.74
1.1	0.1	0.55	10.8	3.8	5.10
3.4	0.4	1.10	14.7	43	20.90
5.4	2.4	1.94	16.5	110	55.49
6.4	3.2	1.84			
7.9	8.9	3.77			
11.5	62	8.25			
16.7	210	43.04			

Surfactant	IC_50_ obs	EC_50_ calc	Surfactant	IC_50_ obs	EC_50_ calc
LIAL 125 BRD			LIAL 125 NRD		
0.4	0.2	0.43	0.4	0.1	0.51
3.1	0.5	0.94	2.7	0.4	0.78
4.8	1.5	1.27	4	1.9	0.99
5.1	1.8	1.36	6.2	3.4	1.77
13.6	4.1	4.11	13.4	13	3.95
18.7	5.5	9.20	19.8	32	7.11

The regression equations for the three parent alcohol types are

Ethoxylated Dodecanol:

7 $$\log{\mathrm{IC}}_{50}\left(V.\mathrm{Fisheri},\mathrm{obs}\right)=1.54\left(0.22\right)\log{\mathrm{EC}}_{50}\left(\mathrm{Fish},\mathrm{calc}\right)+0.47\left(\pm 0.17\right)n=14,,R^{2}=0.798,R^{2}\left(\mathrm{adj}\right)=0.772,s=0.41.F=119.2$$

Ethoxylated ALFOL 12-14:

8 $$\log\;{\mathrm{IC}}_{50}\left(V.\mathrm{Fisheri},\mathrm{obs}\right)=1.43\left(\pm 0.16\right)\log{\mathrm{EC}}_{50}\left(\mathrm{Fish},\mathrm{calc}\right)+0.57\left(\pm 0.15\right)n=10,,R^{2}=0.905,R^{2}\left(\mathrm{adj}\right)=0.893,s=0.29,F=75.7$$

Ethoxylated LIAL 125:

9 $$\log{\mathrm{IC}}_{50}\left(V.\mathrm{Fisheri},\mathrm{obs}\right)=1.52\left(\pm 0.23\right)\log{EC}_{50}\left(\mathrm{Fish},\mathrm{calc}\right)+0.11\left(\pm 0.10\right)n=12,,R^{2}=0.812,R^{2}\left(\mathrm{adj}\right)=0.791,s=0.41,F=42.7$$

Regression analysis for the complete *V. fisheri* data set gives the equation^18^

10 $$\log{\mathrm{IC}}_{50}\left(V.\mathrm{Fischeri},\mathrm{obs}\right)=1.36\left(\pm 0.25\right)\log{\mathrm{EC}}_{50}\left(\mathrm{Fish},\mathrm{calc}\right)-0.32\left(\pm 0.18\right)n=36,,R^{2}=0.778,s=0.41,F=119$$

Although this equation is highly significant, as indicated by the Fisher *F*-value, the statistical quality is poorer than that of the *Daphnia* plot. A possible reason is that in many cases components neglected in the calculation on the grounds of insolubility may in fact be able to contribute to toxicity as a result of not having time to precipitate from solution in the short time scale (5 min) of the test.

By combining with eq 1 and eliminating log EC_50_ (calc) eq 10 can be converted to

11 $$\log\left(1/{\mathrm{IC}}_{50}\right)=1.18\left(\pm 0.22\right)\log P+1.85\left(\pm 0.46\right)$$

It is instructive to compare this equation with published QSARS for toxicity of nonpolar narcotics (IG50 in mol/L) to *V. fischeri* ^18,19^

12 $$\begin{array}{l} \log\left(1/\mathrm{IG}50\right)=1.07\left(\pm 0.04\right)\log P+0.64\\ n=45,R^{2}=0.95,s=0.32 \end{array}$$

13 $$\log\left(1/\mathrm{IG}50\right)=1.17\log P+0.52412\;\mathrm{aliphatic alcohols and ketones},R^{2}=0.969,s=0.38,F=310$$

Although the slopes of eqs 11, 12 and 13 are, within the error limits, identical, as would be expected if the ethoxylated alcohols act as nonpolar narcotics in the *V. fischeri* test, the intercept for the ethoxylated alcohols QSAR (eq 11) is more positive than for the other QSARs, to an extent which cannot plausibly be accounted for by overlap of error limits. We cannot therefore rule out the possibility of a different mode of action for the ethoxylated alcohols. However, an alternative possibility, which we consider more likely, is that the difference in the intercepts results from the less soluble components being in solution to a greater extent than is taken into account in the mixture toxicity calculations.

## Estimation and Application of Solubility Cut-Offs

As stated above, the anomalously low toxicity (high EC_50_) of dodecanol-E16.7 (NRD) to *Daphnia* is at first sight surprising. The calculated EC_50_ values for the pure E16 and E17 ethoxamers, 38 and 41 mg/L respectively, are lower than the observed EC_50_ value of dodecanol-E16.7 (NRD) by a factor of more than 5. Incomplete solubility of the higher ethoxamers seems the most likely explanation. Although increase in E number leads to decrease in log *P*, tending to increase water solubility, above about E3 increase in E number leads to increase in melting point and this, for melting points above ambient temperature, tends to reduce solubility. A similar explanation also seems plausible for dodecanol-E11.5 (NRD) which, apart from dodecanol-E16.7 (NED), is the least well fitted point to both the regression line for the full data set in Figure 1 and the regression line for the Daphnia toxicity of the dodecanol ethoxylates only.

For a single compound, if its solubility is less than its calculated EC_50_ then it is reasonable to assume that it would appear nontoxic if tested–bearing in mind however that there are different EC_50_ values for different end-points and a compound that is too insoluble to exhibit an experimental EC_50_ in an acute test may still be soluble enough to exhibit toxicity in a chronic test. For a component of a mixture, the key issue is whether it is sufficiently soluble to make its calculated toxic contribution to the toxicity of the mixture. If it is not, then the simplest approach is to leave it completely out of the mixture toxicity calculation. However, a more rigorous approach is to use an iterative method incorporating toxic contributions adjusted for solubility cut-offs, and here we derive such a method and apply it to determine whether the low toxicities (high EC_50_) of the outlier dodecanol-E16.7 (NRD) and “near-outlier” dodecanol-E11.5 (NRD), can be rationalized in terms of solubility cut-offs.

Aqueous solubilities of organic compounds are related to their log *P* values and (in the case of solids) their melting points. The relationship can be derived as follows.

The free energy of solution in water Δ*G*_aq_ is given by

14 $$\Delta G_{\mathrm{aq}}=\Delta G_{l/w}\left(+\Delta G_{f}\right)$$

where Δ*G*_l/w_ is the free energy of transfer from the bulk liquid solute to water and Δ*G*_f_, which is only required in the case of a solute which is solid at the temperature of interest, is the free energy of fusion. Δ*G*_f_ can be expressed in terms of the latent heat *L* and entropy Δ*S*_f_ of fusion

15 $$\Delta G_{f}=L-T\Delta S_{f}$$

Since *L* = *T*_m_Δ*S*_f_, where *T*_m_ is the melting point, this can be rearranged to

16 $$\Delta G_{f}=\Delta S_{f}\left(T_{m}-T\right)$$

Therefore the free energy of solution can be written

17 $$\Delta G_{\mathrm{aq}}=\Delta G_{l/w}\left(+\Delta S_{f}\left(T_{m}-T\right)\right)$$

The aqueous solubility *S* can be regarded as the equilibrium constant between the solute in water and the neat solute, and therefore Δ*G*_aq_ can be substituted by −2.303*RT* log *S*. Thus we can write

18 $$\log S=\log K_{w/l}\left(-\Delta S_{f}\left(T_{m}-T\right)/2.303\mathrm{RT}\right)$$

*K*_w/l_ is the partition coefficient between water and liquid solute. It can be linearly related to the octanol–water partition coefficient *P* by a Collander relationship^20^

19 $$\log K_{w/l}=a\log P+b$$

giving the relationship

20 $$\log S=a\log P\left(-\Delta S_{f}\left(T_{m}-T\right)/2.303\mathrm{RT}\right)+b$$

where *S* is the molar solubility and *a* and *b* are constants. Several correlations of this type have been reported,^21–23^ differing only slightly in the values of *a* (close to −1) and *b*. Slight differences are to be expected, since the Collander relationship is strictly valid only within groups of structurally related compounds. In the case of solids, neglect of the - Δ*S*_f_ (*T*_m_ – *T*)/2.303*RT* term leads to *S*′, the supercooled liquid solubility.

A correlation of log *S* against log *P* (for liquid solutes) reported by Hansch et al.^24^ for a series of aliphatic alcohols seems the most appropriate starting point for estimating solubilities of ethoxylated alcohols. However, the Hansch et al. correlation,^24^ which was reported several years before the Leo and Hansch fragment method^1^ was published, is based on calculated log *P* values using an increment of 0.50, as compared with the later value of 0.54, for the contribution of a methylene group to log *P*. The Hansch et al. data set^24^ used in this correlation also contains secondary and tertiary alcohols and multibranched alcohols. We therefore carried out a new regression analysis, using log *P* values recalculated by the fragment method used for the ethoxylated alcohols, for the linear primary alcohols reported by Hansch et al. (*n*-butanol, pentanol, hexanol, heptanol and octanol). We also included solubility data listed by Yalkowsky and Jain^25^ for *n*-nonanol, *n*-decanol and *n*-dodecanol. These data are summarized in Table 5.

**Table 5:** Log *P* and Solubility Data (log *S*, *S* in mol/L) for C4 to C12 Linear Primary Alcohols

*n*-Alcohol, C number	log *P*	-log *S*
4	0.87	–0.026
5	1.41	0.59
6	1.95	1.21
7	2.49	1.81
8	3.03	2.3
9	3.57	3.01
10	4.11	3.62
12	5.19	4.63

Regression analysis of the solubility data for these alcohols gives

21 $$-\log S=1.09\left(\pm 0.02\right)\log P-0.93\left(\pm 0.05\right)n=8,,R^{2}=0.9988,R^{2}\left(\mathrm{adj}\right)=0.9983,s=0.06,F=4228.86$$

For calculation of water solubilities of the alcohols and ethoxylated alcohols considered here we therefore use

22 $$-\log S=1.09\log P\left(+\Delta S_{f}\left(T_{m}-T\right)/2.303\mathrm{RT}\right)-0.93$$

with the Δ*S*_f_(*T*_m_ – *T*)/2.303*RT* term applied only for components with melting point >25 °C.

The temperature of interest, *T*, is taken as 25 °C, 298 °K.

Experimental Δ*S*_f_ values are available for several aliphatic alcohols.^26^ Where experimental values are not available, approximate values can be calculated using Yalkowsky’s formula^27^ for linear organic compounds

23 $$\Delta S_{f}=13.5+2.5\left(L-5\right)$$

where *L* is the number of bonds in the chain. For linear alcohols *L* is equal to the carbon number; for ethoxylated alcohols it is equal to the alcohol carbon number plus three times the number of EO units. Table 6 compares experimental and calculated Δ*S*_f_ values for several linear alcohols, and gives calculated Δ*S*_f_ values for selected ethoxamers.

**Table 6:** Melting Points and Entropies of Fusion

Alcohol C no.	E no.	M. pt. (°C)	Δ*S*_f_ (exp)	Δ*S*_f_ (calc)
10	0	6.9	32	26
12	0	23.8	25	31
14	0	37.8	31	36
16	0	49.3	42	41
18	0	57.9	53	46
12	1	19.5		38.5
	2	17.6		46
	3	16		53.5
	4	18.7		61
	5	22.8		68.5
	6	24.8		76
	7	26.1		83.5
	8	31.4		91

Alcohol C no.	E no.	M. pt	Δ*S*_f_ (exp)	Δ*S*_f_ (calc)
14	1	29^a^		43.5
	2	28.5		51
	3	26.9		58.5
	4	29		66
	5	30.9		73.5
	6	32.8		81
	7	34		88.5
	8	37.5		96

a Estimated.

Melting points are available for linear primary alcohols but only for a few single component ethoxylated alcohols,^28^ as shown in Table 5. Addition of the first few EO units progressively reduces the melting point but as more EO units are added the trend is reversed and the melting point increases. The same pattern is observed for ethoxylated alcohol mixtures with various average degrees of ethoxylation, as described by Rösch.^29^

For higher linear C12 ethoxylates, an estimate of melting point can be made by extrapolating from the experimental data, using formulas derived from melting point/E-number plots given by Rösch^29^ for ethoxylated alcohol mixtures with various average degrees of ethoxylation

up to E11:m.pEn−mp.E(n−1)=64.9⁡log(n/(n−1)) 24

25 above E11:m.p.En−m.p.E(n−1)=28.9⁡log(n/(n−1))

Solubilites of the individual components of the dodecanol ethoxylates can now be calculated, using eqs 22 to 25. The calculations are conveniently carried out by means of a spreadsheet, as illustrated in the Supporting Information document SI2. The calculated solubilities are compared against the calculated (assuming complete solubility) EC_50_ values in Table 7. Also shown are the concentrations of the components (again assuming complete solubility) at the experimental EC_50_s of the mixtures. The ethoxamers up to E8 are all calculated to be completely soluble at their EC_50_s. From E9 to E11 the individual compounds have calculated solubilities below their individual EC_50_s but above their concentrations at the experimental EC_50_ of the mixture. On this basis, all of the ethoxamers up to E11 should contribute fully to the toxicity of the mixture. The E12 and higher ethoxamers are calculated to have solubilites below their nominal concentrations at the EC50 of the mixture, and on this basis should not contribute fully to the toxicity of the mixture.

**Table 7:** Solubilities and Toxicities of Individual Ethoxamers Present in Dodecanols E11.5 and 16.7 (NRD)

			Nominal concentration^b^ (mg/L) at experimental EC_50_ ^c^	
E no^a^.	Ethoxamer EC_50_ (mg/L)	Solubility (mg/L)	E11.5 (NRD)	E16.7(NRD)
0	0.42	3.66	0.02	0.02
1	0.48	4.09	0.29	0.02
2	0.70	6.26	0.58	0.02
3	0.99	9.33	0.87	0.02
4	1.38	13.64	1.16	0.07
5	1.89	19.64	1.45	0.12
6	2.56	27.95	1.74	0.24
7	3.43	27.57	2.03	0.89
8	4.57	5.72	2.32	3.24
9	6.04	1.56	2.61	8.33
10	7.93	0.41	2.9	16.06
11	10.37	0.1	3.19	26.3
12	13.51	0.05	3.48	33.65
13	17.52	0.02	3.77	38.21
14	22.65	0.01	4.06	37.68
15	29.19	0.005	4.35	30.17

a Ethoxamers with E numbers >15 are not included in this table, although they are present in the Dodecanol-E11.5 and -E16.7­(NRD) ethoxylates. Because of their very low solubilities their toxic contributions are negligible.

b Concentration which would apply if the ethoxamer were completely soluble.

c Of the mixture.

It can be seen from Table 7 that the ethoxamers up to E8 are calculated to be fully soluble at the EC_50_s of both Dodecanol-E11.5 and -E16.7­(NRD), but the higher ethoxamers are not completely soluble.

To derive predicted EC_50_ values for dodecanols-E11.5 and -E16.7 (NRD), allowing for incomplete solubility, we start from the QSAR equation for the dodecanol ethoxylates (eq 4). First we rerun the regression with both dodecanol-E11.5 (NRD) and dodecanol-E16.7 (NRD) excluded. This gives the equation

26 $$\log{\mathrm{EC}}_{50}\left(\mathrm{Daphnia},\mathrm{predicted}\right)=0.91\left(\pm 0.06\right)\log{\mathrm{EC}}_{50}\left(\mathrm{fish},\mathrm{calc}\right)+0.21\left(\pm 0.04\right)n=12,,R^{2}=0.957,R^{2}\left(\mathrm{adj}\right)=0.953,s=0.10,F=223.50$$

Then we apply the following algorithm for dodecanol-E11.5­(NRD)

1. Calculate EC_50_(1, fish, calc) for each component *i* of dodecanol-E11.5 (NRD), using the Könemann eq (eq 1). For each component, calculate the number of toxic units as the fraction of that component in the mixture divided by its EC_50*i*_(1, fish, calc) value. Sum the toxic units and take the reciprocal to obtain EC_50_(1, fish, calc), the first estimate of the fish EC_50_ for dodecanol-E11.5 (NRD) predicted by the Könemann eq (eq 1) assuming all components soluble.
2. Use the QSAR eq 26 to derive EC_50_(1, Daphnia, predicted) from EC_50_(1, fish, calc): log EC_50_(1, Daphnia, prediced) = 0.91 log EC_50_(fish, 1, calc) + 0.21
3. Calculate the (hypothetical) concentration *C*_*i*_ of each component at EC_50_(1, Daphnia, pred), and compare each value to the calculated solubility, *S*_*i*_.
4. Calculate EC_50_(2, fish,calc) for the mixture by summing:
  the calculated toxic units t.u. for all components with *C*_*i*_ < *S*_*i*_ and adjusted toxic units calculated as t.u.(adj) = t.u.(*S*_*i*_/*C*_*i*_) for all components with *C*_*i*_ > *S*_*i*_.
5. Repeat 2–4 iteratively until successive iterations converge.
  The workflow for this method of dealing with partial solubility of mixture components is shown in Scheme 2.

**Scheme 2:**
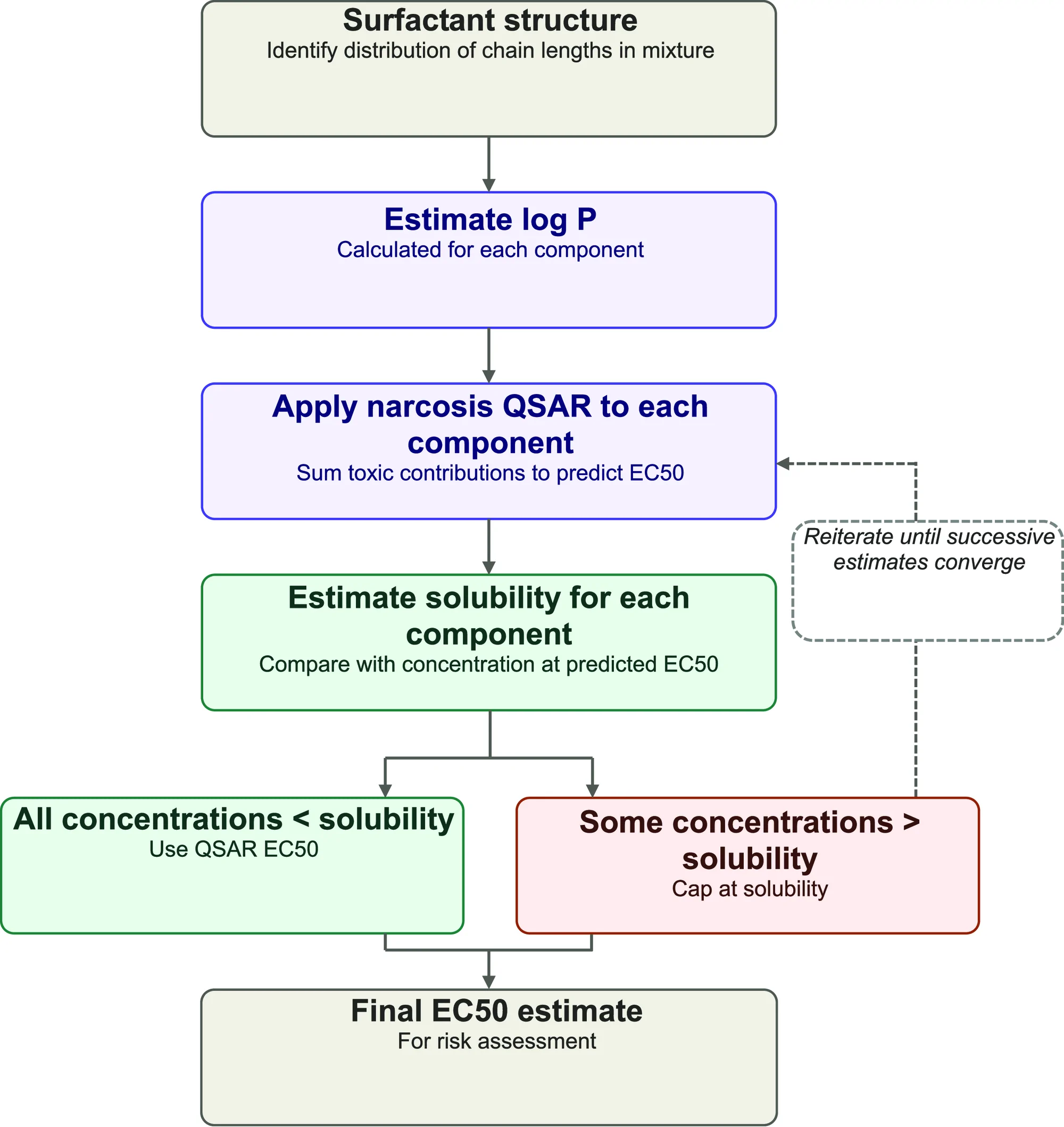
Workflow for Toxicity Prediction of Mixtures of Homologues

Table 8 shows the EC_50_(n, fish, predicted) values calculated for Dodecanol-E11.5­(NRD) after several iterations of the above algorithm. EC50­(fish, calc) converges after 5 iterations to 18.5 mg/L, corresponding to an EC_50_(Dapnia, predicted by eq 20) value of 23.5 mg/L, which is within the 95% confidence range of the experimental value (20–38 mg/L).

**Table 8:** Iterative Calculations of EC_50_ for Dodecanols E11.5 and E16.7­(NRD)

	EC_50_(Fish, calculated), mg/L	
iteration	Dodecanol-E11.5 (NRD)	Dodecanol-E16.7 (NRD)
1 (assumed complete solubility)	8.2	16.4
2	14.8	69.6
3	17.5	106.5
4	18.3	122.7
5	**18.5**	127.7
6		129.1
7		129.4
**8**		**129.5**
**EC_50_(Daphnia, predicted)**	23.5	153.0

We next rerun the regression for the Dodecanol ethoxylates, now including Dodecanol-E11.5­(NRD) but still excluding Dodecanol-E16.7­(NRD). For the Dodecano-E11.5­(NRD) entry we use the solubility corrected EC_50_(fish, calc) value of 23.5 mg/L The resulting regression equation is

27 $$\log{\mathrm{EC}}_{50}\left(\mathrm{Dapnhia},\mathrm{obs}\right)=0.935\left(\pm 0.05\right)\log{\mathrm{EC}}_{50}\left(\mathrm{fish},\mathrm{calc}\right)+0.21(\left(\pm 0.04\right)n=13,,R^{2}=0.965,R^{2}\left(\mathrm{Adj}\right)=0.962,s=0.10,F=301.15...$$

To derive a solubility-corrected prediction for Daphnia toxicity of Dodecanol-E16.7­(NRD) we apply the same algorithm as for Dodecanol-E11.5­(NRD), this time using eq 27. As shown in Table 8, the EC50­(fish, calc) value converges after 8 iterations to 129.4 mg/L, corresponding to an EC_50_(Dapnia, predicted by eq 27) value of 153 mg/L, which is within the 95% confidence range of the experimental value for Dodecanol-E16.7­(NRD) of 130–350 mg/L.

Figure 3 shows a plot of log EC_50_(Dapnhia, obs) vs log EC_50_(fish, calc) for the dodecanol ethoxylates, using the above solubility-corrected EC_50_(fish, calc) values for the E11.5­(NRD) and E16.7­(NRD) ethoxylates.This plot corresponds to the regression eq 21. Dodecanol-E16.7 is not included in the regression. Prediction by extrapolation gives

$$\mathrm{Dodecanol}‐E16.7,\log{\mathrm{EC}}_{50}\left(\mathrm{Daphnia}\right):\mathrm{predicted}\;2.19;,\mathrm{observed}\;2.38$$

**Figure 3:**
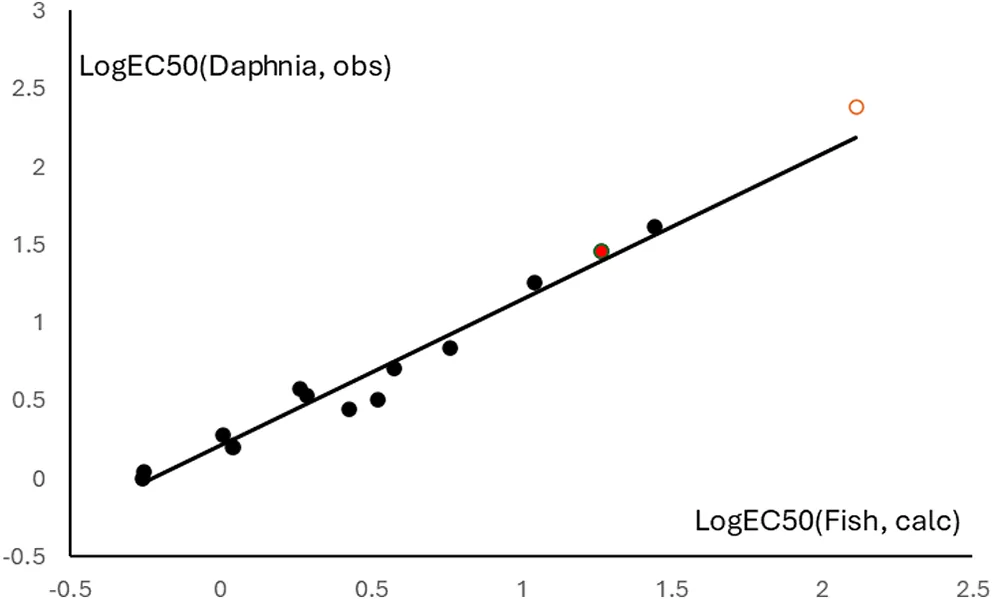
*Daphnia* QSAR for Dodecanol ethoxylates, with solubility-adjusted EC_50_(Fish, calc) values for Dodecanols-E11.5 and 16.7 NRD. Open circle E16.7­(NRD), not included in regression (to avoid undue leverage due to distance from the other points); red circle E11.5 NRD, with EC_50_ calc adjusted for solubility cut-offs, included in regression.

Figure 4 shows the plot for the complete data set. Again, Dodecanol-E16.7­(NRD) is exclued from the regrssion to avoid undue leverage.

**Figure 4:**
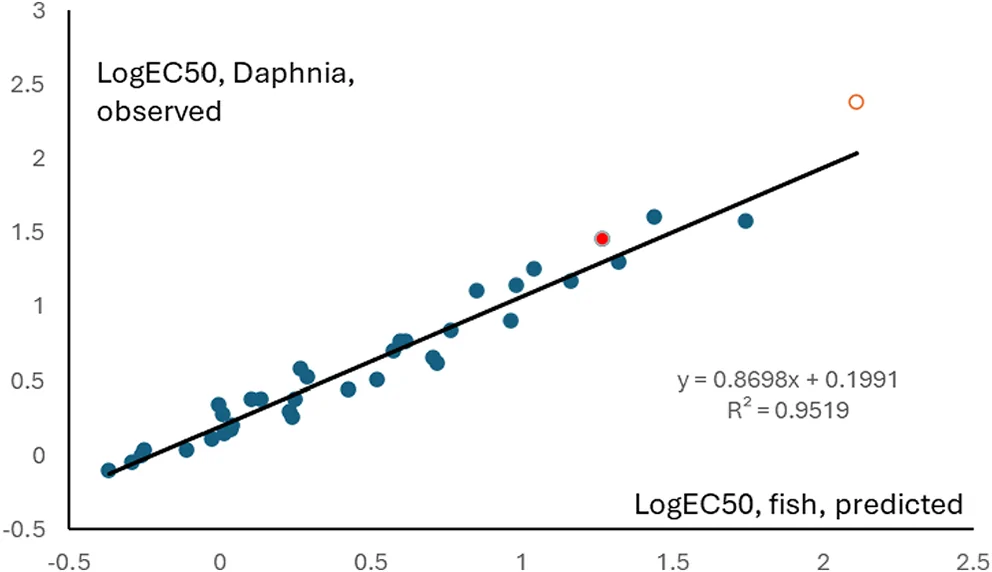
*Daphnia* QSAR for Dodecanol, ALFOL and LIAL ethoxylates, with solubility-adjusted EC_50_(Fish, calc) values for Dodecanols-E11.5 and 16.7 NRD. Black circles: all except Dodecanol E16.7 NRD, excluded from regression to avoid undue leverage; open circle: Dodecanol E16.7 NRD; red circle E11.5, with EC_50_ calc adjusted for solubility cut-offs, included in regression.

The regression equation for the full data set, excluding Dodecanol E16.7 NRD, is now

28 $$\log{\mathrm{EC}}_{50}\left(\mathrm{Dapnhia},\mathrm{obs}\right)=0.87\left(\pm 0.03\right)\log{\mathrm{EC}}_{50}\left(\mathrm{fish},\mathrm{calc}\right)+0.20(\left(\pm 0.02\right)n=13,R^{2}=0.953,R^{2}\left(\mathrm{Adj}\right)=0.951,,s=0.11,F=653.61...\mathrm{Dodecanol}‐E16.7\mathrm{NRD},,\log{\mathrm{EC}}_{50}\left(\mathrm{Daphnia}\right):\mathrm{predicted}\;2.04;\mathrm{observed}\;2.38$$

Although based on several simplifying assumptions and extrapolations, the solubility cutoff algorithm derived here is successful in its application to rationalizing the toxicities of the outlier and “semi-outlier” dodecanol-E16.7NRD and dodecanol-E11.5NRD, respectively.

It should be noted that this solubility cutoff algorithm is based on the assumption that incompletely soluble components with melting points above the ambient temperature separate out individually as crystalline solids. In cases where the relatively insoluble components separate out as oils, these components may tend to precipitate out of solution together, as an oil of mixed composition, when their combined concentration exceeds their composite solubility.

## Conclusions

Surfactants are nowadays often considered as UVCBs (**Unknown** or **Variable** composition, **Complex** reaction products or **Biological** materials) but although the surfactants discussed here are multicomponent mixtures the appropriateness of the UVCB categorization is questionable. Their compositions are known and do not vary significantly for any given commercial surfactant. They are simple mixtures of homologues, isomers and ethoxamers with no complexity other than the number of components, all with the general formula Alkyl-(OCH_2_CH_2_)_n_OH. As shown here, their aquatic toxicity can be modeled in the same way as for single component chemicals.

The findings reported here support the view that calculated log *P* values can be used with Könemann’s general narcosis equation to predict the acute aquatic toxicity of nonionic surfactants by QSAR. However, in applying this equation, together with mixture toxicity calculations, it is important to bear in mind that not all components may be sufficiently soluble to contribute significantly to toxicity. The approach presented in the preceding section represents a first step in evaluating algorithms for taking this into account in QSAR calculations.

The manual method applied here for calculation of log *P* values differs slightly from the present computerized *C* log *P* method although both derive from the same origin. Some comparisons with results from *C* log *P* version 5.9 are dicssussed briefly in the Supporting Information document SI3. Evaluation of other log *P* calculation programs to assess performance against the data set discussed here are under consideration.

The approach we have applied in this paper is very much reductionist. We do not know for sure that our calculated log *P* values are the same as the true log *P* values but we make the simplifying assumption that they at least represent the relative hydrophobicities. Since our log *P* calculations include fragment and factor values (EO unit contribution and position-dependent branching factors) that are not in the published Leo and Hansch method this is less of a confident assumption than it would be for the chemicals that the earlier narcosis QSARs were based on. We know that the biologically relevant partitioning is between water and membranes but, in common with earlier developers of narcosis QSARS, we use log *P*_octanol_ values (in our case calculated), assuming that they represent relative abilities to partition into membranes. Having obtained quite a good correlation we then examine outliers to assess whether they can be rationalized theoretically (as in this case, in terms of assumed partial insolubility) and if so whether they can be brought into line by using whatever relevant information is available to quantitively evaluate (again on a reductionist basis) the proposed reason for their being outliers. Probably the weakest of our simplifying assumptions is that ethoxamers of linear alcohols above C12 can be treated as not contributing to toxicity due to insolubility. Our neglect of these components in the ALFOL and LIAL ethoxylates may be the main reason why the dodecanol-EO regression equation is statistically better than the ALFOL and LIAL regressions. We plan to build on the solubility model presented here by reanalyzing the ALFOL and LIAL data taking into account partly soluble components. We also note that for chronic toxicity end points, where the effect concentrations are lower, the low solubility components can contribute more to the overall toxicity. In light of recent developments in understanding acute to chronic ratios (ACR) for surfactants^30^ there may be scope to apply our solubility cutoff approach in this context.

## Supplementary Material







## Notes

Excel spreadsheet listing all ethoxamer distributions and toxicity data (XLSX)
Excel spreadsheet showing solubility cutoff algorithm for by Dodecanol-E11.5­(NRD) and Dodecanol-E16.7­(NRD) (XLSX)
Brief comparison of manual and computerized (*C* log *P*) methods for log *P* calculation (PDF)
