# Supplementary material for: QSAR Modeling of Acute Aquatic Toxicity of Ethoxylated Alcohol Mixtures, and Estimation of Solubility Cut-Offs
Source: Chem Res Toxicol. 2026 Aug 28;39(9):1648–58. doi: 10.1021/acs.chemrestox.5c00561 (PMC13632904; doi:10.1021/acs.chemrestox.5c00561)
Supplement: Supplementary file 1 [file tx5c00561_si_003.pdf]

SUPPORTING INFORMATION

QSAR MODELLING OF ACUTE AQUATIC TOXICITY OF ETHOXYLATED ALCOHOL MIXTURES, AND ESTIMATION OF SOLUBILITY CUT-OFFS

David W. Roberts[†]*, M. Teresa García[‡], Isabel Ribosa[‡]

† School of Pharmacy and Biomolecular Sciences, Liverpool John Moores University, Liverpool L3 3AF

‡ Department of Surfactants and Nanobiotechnology, Institute for Advanced Chemistry of Catalonia (IQAC-CSIC), 08034 Barcelona, Spain

*Corresponding author, email d.w.roberts@ljmu.ac.uk

SI1. Excel spreadsheet listing all ethoxamer distributions and toxicity data

SI2. Excel spreadsheet showing solubility cut-off algorithm, illustrated by Dodecanol-E11.5(NRD) and Dodecanol-E16.7(NRD)

SI3. Brief comparison of manual and computerised (ClogP) methods for logP calculation

Supplementary information 3. Notes on the calculation of logP

The manual method applied here for calculation of logP values[1] differs in a few respects from the present computerised ClogP method although both derive from the same origin, and we have made some comparisons with results from ClogP version 5.9:

CH_2 increment

In the manual method[1], the derived fragment value for a CH_2 unit is 0.66 (the combination of two rounded off H fundamental fragment values of 0.23 each and one rounded off C fundamental fragment value of 0.20), which with the addition of a bond factor F_b (-0.12) gives 0.54 as the logP increment for a CH_2 group. In the computerised ClogP method all the fundamental fragment values are added together rather than using derived fragment values. The fundamental fragment values used for H and C are 0.227 and 0.195 respectively. Combining these and adding the bond factor (-0.12, the same as in the manual method) gives a CH_2 increment value of 0.529, i.e. 0.011 less than calculated by the manual method. This difference would not significantly affect the calculated logP values for the compounds with C12-C15 alkyl chains.

Ethyleneoxy (EO) increment

The computerised ClogP method does not use a derived EO increment value. Instead one -O- , four H and 2 C fundamental fragment values plus three bond factor values and one proximity factor value are all added to the total to be summed. This results in a logP decrease of 0.176 going from $ROCH_2CH_2OH$ to $ROCH_2CH_2OCH_2CH_2OH$ (logP values calculated by ClogP 5.9) correponding to a derived fragment value of -0.176 for a EO unit. In contrast, when the logP values for these two compounds are calculated manually by the orginal method[1], (i.e. not using a derived fragment value for the EO group but treating it as a combination of two CH_2 groups and one ether oxygen and applying bond and proximity factors) the EO contribution is calculated to be slightly positve (0.09 based on the rounded

CH_2 fragment value of 0.66, or 0.06 based on the C and H fragment values of 0.195 and 0.227 respectively). These differences derive basically from the way in which proximity between vicinal H-polar groups (in this case -O- and -OH) is treated. In the manual method (and, we believe, in early versions of the computerised ClogP method) the fragment values of the two H-polar groups are added together and multiplied by -0.26 to give a proximity factor. In ClogP version 5.9 this still applies to vicinal proximity between -O- and -OH (fragment values -1.82 and -1.64 respectively), giving a proximity factor of +0.90, but for vicinal proximity between two -O- units the proximity factor is calculated as +0.706, which corresponds to multiplying the sum of the two vicinal -O- fragment values by 0.194.

It is recognised that EO groups can exhibit different hydrophobicity/hydrophobicity values depending on temperature and on the molecular environment[7] , but for nonionic surfactants based on ethoxylated alcohols at ambient temperatures the literature[2-6] supports -0.10 for the derived fragment value of the EO group. For low degrees of ethoxylation the difference of 0.076 per EO group between the manual and the ClogP (version 5.9) methods would not significantly affect the calculaled logP values, but the difference would become more significant with higher degrees of ethoxylation.

Branching factors

The computerised ClogP method, like the original manual method, applies a factor for branching in an alkyl chain, the value being -0.13 or -0.22 (for branching at a polar substiuent point) irrespective of the length of the branch. Consequently, all monobranched isomers of a LIAL alcohol R**R**CHCH$_2$OH and of each of their ethoxylates would have the same calculated logP value. Here we apply the position dependent branch factor (PDBF), originlly derived for modelling fish toxicity of individual components of linear alkyl benzene sulfonate surfactants[7,8] and subsequently found to be applicable not only to other surfactants[4,6,9-12] and to other surfactant endpoints (e.g. critical micelle concentration[11]) but also to non-surfactants. In the present study only the LIAL-based ethoxylates would be affected by this difference in how branching is modelled.

Refereneces

1. Leo A J and Hansch C. 1979. Substituent Constants for Correlation Analysis in Chemistry and Biology. Wiley, New York.
2. Roberts D W. 1991. QSAR issues in aquatic toxicity of surfactants. Sci. Total Environ. 109/110: 557-568.

3. Roberts D W and Marshall S J.1995. Application of hydrophobicity parameters to prediction of the acute aquatic toxicity of commercial surfactant mixtures. *SAR and QSAR in Environmental Research. 4*:167-176.
4. Roberts D W, Marshall S J and Hodges G. 1996. Quantitative structure-activity relationships for acute aquatic toxicity of surfactants. *World Surfactants Congr., 4th*, **4**:202-212.
5. Roberts D W. 2004. Environmental Risk Assessment of Surfactants: Quantitative Structure-Activity relationships for Aquatic Toxicity. In U Zoller Ed. *Handbook of Detergents: Part B, Environmental Impact.* Dekker, New York. 271-298.
6. Roberts J F, Marshall S J and Roberts D W. 2007. Aquatic toxicity of ethoxylated and propoxylated alcohols to Daphnia magna. *Environmental Toxicology and Chemistry, 26*, 68–72.
7. Roberts DW. 1988. Aquatic Toxicity of Linear Alkyl Benzene Sulphonates (LAS) – A QSAR Analysis. In QSAR 88 (91 – 98) Proceedings of the Third International Workshop on Quantitative Structure–Activity Relationships in Environmental Toxicology, May 22 – 26 1988, Knoxville, Tennessee. Ed. J E Turner, M W England, T W Schultz and N J Kwaak. Available from the National Technical Information Sevice, US Dept of Commerce, Springfield, Virginia 22161, USA.
8. Roberts DW. 1989. Aquatic Toxicity of Linear Alkyl Benzene Sulphonates (LAS) – A QSAR Analysis. Jorn. Com. Esp. Deterg, 20 (1989) 35 – 43.
9. Rosen M J, Fei L, Zhu Y-P and Morrall S W. 1999. The Relationship of the Environmental Effect of Surfactants to Their Interfacial Properties, *J. Surf. Deterg*. *2*, 343-347.
10. Roberts DW. 2000. Aquatic Toxicity - Are Surfactant Properties Relevant? *J. Surf. Deterg*. **3**, 309-315.
11. Roberts DW. 2002. Application of Octanol/Water Partition Coefficients in Surfactant Science.: A Quantitative Structure-Property Relationship for Micellisation of Anionic Surfactants. *Langmuir* **18**, 345-352.
12. Roberts D W. 2004. Environmental Risk Assessment of Surfactants: Quantitative Structure-Activity relationships for Aquatic Toxicity. In U Zoller Ed. *Handbook of Detergents: Part B, Environmental Impact.* Dekker, New York. 271-298.
